# Navigating Pain in Rheumatology: A Physiotherapy-Centric Review on Non-pharmacological Pain Management Strategies

**DOI:** 10.7759/cureus.51416

**Published:** 2023-12-31

**Authors:** Avilash Mohapatra, Sneha Patwari, Mukta Pansari, Srikanta Padhan

**Affiliations:** 1 Department of Surgery and Physiotherapy, All India Institute of Medical Sciences, New Delhi, New Delhi, IND; 2 Department of Physiotherapy, Regional College of Paramedical Health Science, Guwahati, IND; 3 Department of Physiotherapy, Jai Prakash Narayan Apex Trauma Center, All India Institute of Medical Sciences, New Delhi, New Delhi, IND; 4 Department of Community and Family Medicine, All India Institute of Medical Sciences, Raipur, Raipur, IND

**Keywords:** hydrotherapy, psychosocial pain, physiotherapy, rheumatic disease, pain

## Abstract

Rheumatism is a broad term for the painful afflictions of the musculoskeletal system, which include a variety of symptoms ranging from vague pain or aching to profound disability. This article explores the imperative role of physiotherapy in navigating pain within the field of rheumatology, providing a comprehensive review of non-pharmacological pain management strategies. A literature search of PubMed, Web of Science, Scopus, and Cochrane databases was conducted, employing keywords like "Pain," "Rheumatic disease," and "Physiotherapy," with the review emphasizing recent English studies, particularly randomized trials, meta-analyses, and systematic reviews over the last 10 years, to consolidate evidence on the efficacy of physiotherapy interventions for individuals with rheumatic disease. Pain, a significant challenge for individuals with rheumatic diseases, is often intense and persistent, associated with subsequent physical disability, but employing a holistic approach encompassing drugs, physical therapy, and patient education can yield substantial benefits in managing these painful conditions. In addition to pharmacological interventions, management strategies incorporate a non-pharmacological approach, encompassing rehabilitation and physical therapy in alignment with the International Classification of Functioning, Disability, and Health (ICF) model. The patient and physiotherapist collaborate to develop a goal-oriented treatment plan, utilizing modalities like heat, cold, electrotherapy, and hydrotherapy for pain management, progressing to mobility enhancement, posture re-education, and activities focused on a range of motion and muscle strengthening.

## Introduction and background

Rheumatism, one of the terms used in medicine the most, often known as a rheumatic disorder, is an umbrella term for any painful condition affecting the musculoskeletal system, which includes the skeleton, muscles, connective tissues, joints, and soft tissues around them [1]. However, the term "rheumatism" is ambiguous; for some people, it might refer to any vague pain or aching, while for others, it evokes images of a wheelchair-bound patient who is paralyzed.

The prevalence of rheumatic disease and related disability is rising in this era of advanced health services. Approximately 75% of patients with rheumatoid arthritis (RA) and systemic lupus erythematosus (SLE) are females. A higher proportion of women suffer from these two rheumatic disorders [2]. The detrimental effects of RA on people and society as a whole, including reduced quality of life, lost productivity, and higher healthcare expenditures, have been demonstrated by epidemiological research on the condition. The quality of life is particularly impacted among women with SLE and RA, according to evidence [3].

Mostly, synovial joints are affected by rheumatic disorders, wherein the most prevalent ones include RA, ankylosing spondylitis, and osteoarthritis [4]. Degradation of "articular cartilage and subchondral remodeling" is a hallmark of osteoarthritis; "chronic systemic inflammatory disease that predominantly affects synovial joints, especially small joints in the hand and foot" is the definition of rheumatoid arthritis. A high degree of enthesopathy, a "tendency of familial aggregation," "chronic inflammation of the spine (including sacroiliac joints)," and an association with human leukocyte antigen (HLA)-B27 are the characteristics that distinguish ankylosing spondylitis [5].

One way to RA is by using the International Classification of Functioning, Disability and Health (ICF). According to the ICF model, medical treatment focuses mostly on the biological function and the health state, while rehabilitation therapies primarily address participation and activity [6].

Analgesics, non-steroidal anti-inflammatory drugs (NSAIDs), and disease-modifying anti-rheumatic drugs (DMARDs) are among the pharmacological treatments for rheumatic illnesses [7]. Moreover, research has demonstrated the benefits of using glucocorticoids as an adjuvant with DMARDs in the treatment of rheumatic disorders [8]. Physical modalities, orthoses or assistive devices, therapeutic exercise to improve strength, endurance, mobility and patient education are all part of the rehabilitation care for rheumatic disorders [9]. The patient's condition should determine the course of treatment.

The field of rehabilitation medicine has emerged since "rheumatology" first appeared. It provides treatment for a range of conditions, including rheumatic diseases, neurological abnormalities, and surgical amputations. It includes the specializations of physiotherapy and occupational therapy [10].

This article aims to provide a thorough exploration of non-pharmacological pain management strategies in rheumatology, with a specific focus on the integral role of physiotherapy, offering a comprehensive understanding of how these strategies can effectively navigate and address pain, enhance mobility, and improve the overall functional outcomes for individuals with rheumatic diseases.

## Review

Methodology

To conduct a comprehensive review of non-pharmacological pain management strategies in rheumatology, we conducted a systematic literature search between July 1, 2023, and October 31, 2023. Our search focused on online databases, including PubMed, Web of Science, Scopus, and Cochrane. The search strategy incorporated keywords such as “Pain,” “Rheumatic disease,” “Hydrotherapy,” “Psychosocial pain,” and “Physiotherapy”. We limited the search to studies published in the English language within the last 10 years. Inclusion criteria comprised randomized trials, meta-analyses, and systematic reviews, while case reports, editorials, and cohort studies were excluded. The initial results were evaluated by group members, ensuring relevance to the topic and adherence to the predefined criteria. Our review synthesizes the latest evidence on non-pharmacological pain management strategies in rheumatology, with a focus on physiotherapy interventions, including rehabilitation, exercise therapy, strength training, manual therapy, hydrotherapy, and physical modalities, aiming to provide a holistic understanding of their effectiveness in enhancing mobility, alleviating pain, and improving functional independence in patients with rheumatic diseases.

Rheumatic disease and physiotherapy

**Figure 1:**
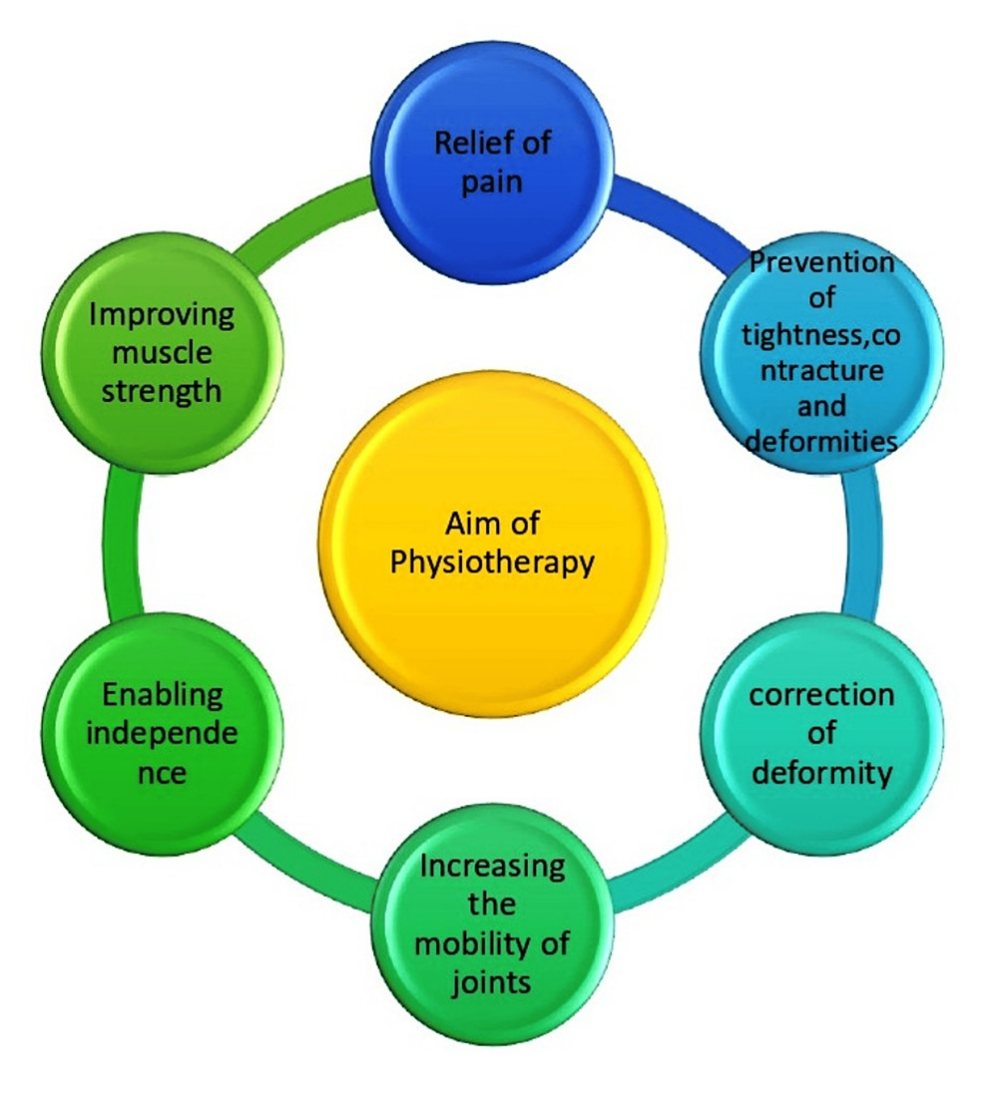
Aim of physiotherapy in rheumatic diseases. The figure is the authors' own creation.

The role of the physiotherapist (Figure 1) in the treatment of patients with rheumatic disease is to collaborate with the patient in order to help them reach and sustain their highest level of independence and function [11]. This will require many patients to participate actively in their social, professional, and familial life. A significant amount of physiotherapy time is spent managing rheumatic disorders, and hospitals that concentrate on rheumatic disease have physiotherapy departments dedicated solely to this area of physiotherapy. Traditionally, physical and occupational therapists have treated patients using non-pharmacologic techniques [12]. The following include walking aids, braces, splints, orthoses for the feet, electrotherapy, patient education, therapeutic physical activity, and assistive devices for activities of daily living (ADL). There is currently little information regarding the use of braces, foot orthoses, and other assistive devices for ADL by patients with arthritis. This is especially true in the population with arthritis [13].

During comprehensive patient examinations, physiotherapists in rheumatology will determine the physical effects of a patient's condition and the extent to which it impacts the person's ability to function, such as their posture, mobility, etc. The therapist assesses the patient's musculoskeletal system to get the baseline of their current condition and take into account additional bodily systems, such as the neurological and cardiovascular systems. Also make a decision for any specific equipment needs, such as the need for splints, walking assistance, or altered shoes. The inevitable part of assessment is learning about the patient's current coping strategies and self-management. Following that, the therapist determines that physiotherapeutic methods are necessary [14].

The patient and the physiotherapist collaborate to create a goal-oriented treatment plan after the physiotherapist reviews the assessment results. This could involve using heat, cold, electrotherapy, and hydrotherapy to treat pain. Following that, the patient can advance to additional treatment modalities such as mobility improvement, posture re-education, and range of motion and muscle strengthening activities [15].

Pain in rheumatic diseases

Pain, another major problem for people with rheumatic diseases, is associated with subsequent physical disability. Patients with rheumatic disease go through pain that can be intense, persistent, and disabling. The pain is based on many factors, which can have central and peripheral components [16]. Since many conditions can cause musculoskeletal pain, patient evaluation and management must start with a complete assessment that identifies plausible causes and measures objective findings against subjective complaints. It is important to consider the likelihood of concomitant pain of central origin, particularly in individuals with a history of RA, and to administer the proper medication [17]. By employing a holistic therapy plan of drugs, physical therapy, and patient education, significant benefits can often be obtained in this group of painful diseases. There is growing evidence that augmented pain regulation, as found in fibromyalgia, is significant in RA and osteoarthritis [18]. These findings find application in optimal management strategies in rheumatic diseases.

In order to assist patients in using their joints as comfortably as possible, joint protection techniques are an educational intervention that can help them feel less discomfort and use less energy. Energy-saving measures also apply to assistive technology and splints. The most effective educational strategy for altering and sustaining the ideal behavioral change is a key question in determining whether the behavior change leads to improved symptoms.

Exercise therapy

In addition to prescription medication treatment, physical therapy has long been a mainstay of the management of inflammatory rheumatic diseases; nevertheless, its inclusion in treatment guidelines for certain disorders is limited [19]. It should be noted that both studies were published in the mid-1990s and physicians’ perceptions of the value of exercise and other non-pharmacologic modalities might have changed over the years. Recent data from a survey of rheumatologists in Ontario, Canada, suggests that this theory is validated. Of those surveyed, 95.6% "strongly agreed" or "agreed" that exercise was useful in the management of RA [20]. In the last two decades, the discovery of myokines has led to the physiological correlations of the anti-inflammatory effect of physical activity. There is sufficient data from many randomized controlled trials for spondylarthritis and RA to support well-founded recommendations [21]. Exercise has potential psychological benefits in addition to its physical ones. It enhances capillarization and tropism in the muscles while lowering hypoxia [22]. Additionally, it stimulates the release of growth hormone and endorphins, raises serotonin levels in the brain, and activates adrenergic pain-inhibitory processes.

Increasing pain and subsequent stiffness as the patient mobilizes are warning signs that too much exercise is being provided [23]. Periods of activity lasting five or 10 minutes at one- or two-hourly intervals are likely to be better tolerated than more intensive sessions of exercise once or twice daily.

Aerobic exercises

In one study, three low-intensity aerobic exercise regimens (15, 25, and 35 minutes) were followed by women with rheumatoid arthritis three times a week for a duration of 12 weeks. Here, they used a control group that wasn't in training. Every workout group saw improvements in their joint counts, aerobic capacity, and exercise duration. The subjects reported less tiredness and joint pain as well as an improvement in their everyday activities. For those with severe limits from rheumatoid arthritis, as little as 15 minutes of exercise three times a week is sufficient to increase aerobic capacity. Exercise sessions lasting up to 35 minutes can be helpful [24].

Cardiovascular fitness training can be performed in various ways and may include elements of simultaneous ROM exercise and muscle training. Scientific evidence exists for fitness training in the form of biking, brisk walking, aquaerobics, and different types of circuit training. Exercise modes can therefore be customized to the patient's preferences; nonetheless, for improving cardiovascular fitness, precise dosage is considerably more crucial than mode [25].

The patient may receive information from the physiotherapist about their condition and guidance on long-term self-management. The patient can then adjust their workout regimen based on their illness activity. Education about family and careers is also an important part of the physiotherapist’s role.

Strengthening exercises

According to a review article, it was concluded that K-taping and strengthening exercises have a considerably significant effect on managing the symptoms in people with RA [26]. Strengthening exercises, K-tape, paraffin wax baths, and moist heat therapy were used to alleviate infected joints' pain, mobility, and muscular power. Parameters such as hand movements, grip strength, and physical activities of daily life showed significant improvement.

In another systematic review and meta-analysis, resistance training was found to reduce joint count, functional capability impairment, and disability [27]. Additionally, because RA patients are at an elevated risk for osteoporotic fractures and related pain, it also enhances neuromuscular performance, which may reduce the risk of falls and fractures. In addition to having anti-inflammatory properties, it also lowers cardiovascular risks. However, there isn't much research on how to maintain the benefits of resistance training over the long run. Improvements in muscle strength following an intervention, according to some authors, only persisted for a short while. As a result, research suggests continuing an organized physical activity program following an intervention is essential for reaping long-term advantages.

Reduced flexibility, low endurance and spasm, spinal muscular shortening, and weaker abdominal muscles are all consequences of a sedentary lifestyle. Sedentary lifestyles and insufficient exercise are two factors that lead to back discomfort. Reduced flexibility of the postural muscles leads to the typical protraction of the shoulder and hyperlordosis. An exercise program should address the postural muscles of the spine, together with the abdominal muscles and the muscles of the shoulder girdle.

Manual therapy

Among the pain complaints in rheumatic disorders, low back pain and neck pain are the most frequently treated disorders by manual therapy. Manipulation has been shown to decrease joint pain and normalize function. Although the exact mechanism of action is unknown, current hypotheses suggest that pain is caused by an imbalance in muscle activity, which can be relieved by modulation by reflexive actions. Also, it has been suggested in a systemic review that manual therapy in adjunct to exercise can be implemented in the treatment of OA [28].

The degree of muscular flexibility is determined by body temperature, the environment, and the degree of muscle training. There are several advantages to an initiative to increase flexibility. Reduced risk of injury, reduced soreness following exercise, less contracture, reduced joint pain, and reduced myofascial pain are some of the benefits. Ballistic stretching, static stretching, passive stretching, and proprioceptive neuromuscular facilitation are some of the stretching techniques.

Hydrotherapy

The Community Rehabilitation Network (CRN) originally launched the community-based water exercise program (CBWEP) in Hong Kong in 1997 for individuals with rheumatic conditions. People with rheumatic disorders can receive physical, psychological, and social support through the CRN program [29].

According to one review, only active contraction has been credited with increasing muscle strength [30]. Exercises in the horizontal plane of limbs supported by the buoyancy of water can be done easily by people with weak muscles or painful joints. The benefits of exercise programs can be described as improving general fitness, increasing joint mobility, and reconditioning.

One type of activity utilized in physiotherapy to treat patients with RA and SLE is hydrotherapy [31]. People with rheumatic disease can exercise with less weight bearing over their damaged joints in a relatively safe and comfortable environment by using the effects of buoyancy and warmth through aquatic exercise. Hydrotherapy has generally been demonstrated to be advantageous in terms of increased functional mobility, reduced discomfort and muscular spasm, and enhanced joint range of motion and muscle power [32].

Physical modalities

When compared to all other modalities for treating musculoskeletal issues, heat and cold therapy has the largest body of literature. Usually, heat therapy involves applying hot packs, electrical pads, thermal packs, paraffin wax, or water baths topically to the patient's skin. Heat can be transferred through three different methods: radiation (infrared light), conduction (hot packs, water, or paraffin), or conversion (using ultrasound or diathermy). Local or superficial heat can be produced by radiant and conductive heat sources, while deep heat can be produced by short-wave, microwave, diathermy, and ultrasonography. Cold therapy is commonly applied topically on the sore area using ice packs, chemical packs, reusable gel packs, or ice massage. It has been proposed that heat therapies are more effective in treating non-inflammatory rheumatic disorders by improving joint flexibility and reducing fluid viscosity [33]. On the other hand, since cold therapies lessen inflammation and articular cartilage degradation, they are recommended for the treatment of active arthritis.

Traction, which can be administered manually or with the aid of traction equipment, reduces pain and enhances angular movement by extending the articular and peri-articular structures. The cervical spine and its appendages are treated with manual traction. The patient's own body weight may be used with slings for bigger joints, such as the hip. Though they should only be used under strict supervision and with weight adjustments, mechanical appliances help with cervical spine traction. For low back pain, whether or not radicular symptoms are present, mechanical traction has been used [34].

Electromodalities

Electrical stimulation of the nerves, muscles, or both is known as electrotherapy. Surface electrodes (transcutaneously) are the primary method of applying electricity. Percutaneous needle electrode application is used in certain procedures, such as electro-acupuncture and dorsal column stimulation. By passing direct current (DC) across the painful area, galvanic direct current (DC) suppresses both nociceptors and slow fibers that mediate pain. The effect of stimulation on peripheral nerve fibers is weaker than that of modulated current. Acute radicular pain and inflammation of peri-articular tissues, such as ligaments and tendons, are the two main indications for galvanic DC therapy. Galvanic electrotherapy is used to increase the absorption of topical medications, particularly anti-inflammatory ones, by improving the transport of ionized substances through the skin (via the process of iontophoresis).

Partial, afferent, and efferent neurons are greatly affected by modulated DC or AC (alternative current), which is often delivered as rectangular impulses. When it comes to controlling pain, it uses a different mechanism than galvanic DC: it inhibits pain-related potentials at the spinal and supraspinal levels. Even though the precise mechanism at work is yet unknown, this mechanism is often known as "gate control." In essence, pain can be somewhat inhibited by electrical stimulation of the myelinated, fast-conducting peripheral nerve fibers. It is not always necessary for the current to travel through the painful area because applying electrodes extra- and contralaterally might also lessen pain in that area [35]. The notion of gate control is applicable to both traditional techniques of stimulating electrotherapy and the more recent type known as transcutaneous electrical nerve stimulation (TENS), which employs 0.2 ms sharp impulses within the 1±150 Hz frequency range. TENS devices have the benefit of being portable, allowing patients to use them anytime and anywhere they are needed due to their tiny size. While a minimum application time of thirty minutes is recommended, there is no need to restrict daily use.

## Conclusions

The rise in the prevalence of rheumatic diseases calls for a more holistic approach towards its management of pain by helping in preventing the complications and disability that come with it, and also enabling more functional independence. Since it is a complex spectrum of musculoskeletal conditions, its management asks for a physiotherapeutic approach alongside pharmacological treatment. Diverse modalities, such as aerobic exercise, hydrotherapy, and manual therapy, have emerged as a cornerstone in alleviating pain, restoring function, and enhancing the quality of life in individuals. Continued research and an integrative approach are of utmost importance in dealing with the multidimensional aspects of rheumatic diseases and enhancing the health of those affected.
